# Depressive Symptoms and Healthy Behavior Frequency in Polish Postmenopausal Women from Urban and Rural Areas

**DOI:** 10.3390/ijerph18062967

**Published:** 2021-03-14

**Authors:** Mariusz Gujski, Dorota Raczkiewicz, Ewa Humeniuk, Beata Sarecka-Hujar, Artur Wdowiak, Iwona Bojar

**Affiliations:** 1 Department of Public Health, Medical University of Warsaw, 02-097 Warsaw, Poland; mariusz.gujski@doktor.pl; 2 Institute of Statistics and Demography, Collegium of Economic Analysis, SGH Warsaw School of Economics, 02-513 Warsaw, Poland; dorota.bartosinska@gmail.com; 3 Department of Pathology and Rehabilitation of Speech, Medical University of Lublin, 20-059 Lublin, Poland; humewa@wp.pl; 4 Department of Basic Biomedical Science, Faculty of Pharmaceutical Sciences in Sosnowiec, Medical University of Silesia in Katowice, 41-200 Sosnowiec, Poland; 5 Diagnostic Techniques Unit, Medical University of Lublin, 20-059 Lublin, Poland; wdowiakartur@gmail.com; 6 Department of Women’s Health, Institute of Rural Health, 20-090 Lublin, Poland; iwonabojar75@gmail.com

**Keywords:** depression, postmenopausal women, rural, urban, healthy behavior

## Abstract

The objective of this study was to determine whether the severity of depressive symptoms was linked to healthy behaviors in Polish postmenopausal women and whether the strength of the link differed between women living in urban versus rural settings. The study was conducted in 2018 in the Lublin region of Poland and included 396 postmenopausal women (239 living in rural areas and 157 in urban areas). The severity of depressive symptoms was evaluated by the Beck Depression Inventory (BDI) and the frequency of healthy behaviors was assessed using the Inventory of Healthy Behaviors. Postmenopausal women living in rural areas underwent menopause significantly earlier, were more often widowed, more often obese, more often less educated, and less likely to have never married when compared to those living in urban areas. Importantly, rural postmenopausal women endorsed more depressive symptoms (*p* = 0.049). There was a negative correlation between the severity of depressive symptoms and age in urban postmenopausal women (r = −0.174, *p* = 0.029), but this was not evident in rural women (r = −0.034, *p* = 0.600). The frequency of healthy behaviors was significantly lower in rural postmenopausal women, especially with respect to nutritional habits. A positive correlation was found between the frequency of healthy behaviors and the level of education in both sets of women (*p* = 0.034 and *p* = 0.045, respectively). To summarize, we found a significant link between healthy behaviors and depressive symptoms in postmenopausal women. We also found that this link was more evident in rural than in urban women.

## 1. Introduction

Postmenopause is a period that begins approximately 12 months after a woman’s last menstrual period and is associated with a specific profile of sex hormones [1]. It is estimated that by 2030, 1.2 billion women will be postmenopausal, which represents as many as 47 million women each year [2,3]. During menopause, a woman experiences many physical and mental changes. Some of them are normal consequences of both menopause and aging, whereas others may be a beginning of developing disease. Good physical and mental health during the postmenopause period is an important component of healthy and successful aging and has many benefits, for both an individual and society on the whole [2].

Depressive disorders are a common health problem in the postmenopausal period, with typical symptoms such as sadness, tearfulness, irritability, emotional liability, and poor concentration [4]. Susceptibility to depression during this period is associated with some women’s increased sensitivity to changes in hormonal status. Factors unrelated to the menopausal transition, including comorbidities, sleep problems, and stressful life events, also play an important role. On the other hand, variables such as ethnicity, culture, tradition, prior history of depression, body mass index, level of education, and marital status are also associated with postmenopausal depression [5,6].

The data from longitudinal studies show that the risk of depressive symptoms in women increases with age. In the early phase of the menopausal transformation, it increases from 1.30 to 1.55 times. On the other hand, in the postmenopausal period, the probability of depression is from 1.71–2.89-fold higher than before menopause [7,8,9]. In turn, the risk of developing Major Depressive Disorder (MDD) increases in the postmenopausal period by two to four times. This, however, only concerns women with a history of such disorders [3].

Analysis of a sample of Polish perimenopausal women [10] showed depressive symptoms in 33–45% of them. It was demonstrated that 30% of women suffered their first episode of depressive symptoms in the four years prior to their last menstruation. Depressive symptoms were reported to be more severe in postmenopausal women, with over one-third of women meeting criteria for mood disorders [11,12].

On the other hand, women on hormonal therapy were reported to show only mild depressive symptoms (13.5%) [13].

Depression, apart from having a negative impact on psychosocial well-being, interpersonal and family relationships, social, professional as well as sexual functioning, affects physical health. While it is difficult to show the relationship between depression and deterioration in physical function, some evidence supports this correlation in older populations [14].

Previously, it has been shown that depression in postmenopausal women is positively associated with, among other diseases, cardiovascular diseases, metabolic syndrome, osteoporosis, and fractures [5,15].

Healthy behaviors, such as a balanced diet, good hygiene, exercise, adequate sleep, avoidance of alcohol and cigarettes, stress modulation, and regular medical exams, appear indicated. They not only reduce the risk of developing the disease but also have a positive effect on mental well-being [16,17].

The available data demonstrate a relation between depression and abnormal health behaviors [18,19]. It has been suggested that depression contributes to a loss of in-health maintenance [20].

It has also been noted that one’s place of residence influences various aspects of women’s health during menopause. Some literature has indicated health discrepancies between rural and urban inhabitants. The factors underlying these disparities include socioeconomics (education, access to skilled work) and access to health services [21,22].

The aim of our study was to assess the severity of depressive symptoms in postmenopausal women from urban and rural areas and determine their link to health-related behaviors.

## 2. Materials and Methods

### 2.1. Study Group

This study was the second part of a research project devoted to the severity of depressive symptoms in postmenopausal women living in urban and rural areas. The first part of the project assessed the impact of serum thyroid-stimulating hormone (TSH) and the plasma concentration of vitamin D concentrations as well as the presence of metabolic syndrome on the severity of depressive symptoms in Polish postmenopausal women from urban and rural areas [23].

The current study was conducted in 2018/2019 within the Lublin region, Poland, and included 396 postmenopausal women (239 living in rural areas and 157 living in urban areas). The inclusion criteria were age 45–65 and at least 12 months from the last menstruation. The exclusion criteria were active cancer within five years before recruitment; medical history of mental disease; addiction to drugs of abuse and/or alcohol; diagnosed dementia; current or past use of hormone replacement therapy (HRT) and severe menopausal symptoms on the Kupperman scale as identified by the Montreal Cognitive Assessment. Only women with a score of at least 26 were included in the study.

Participation in the survey was voluntary, and informed consent for participation was obtained from all volunteers.

The study was approved by the Ethics Committee of Institute of Rural Medicine in Lublin, Poland (protocol code IMW 07/2015, date of approval 15.09.2015)

### 2.2. Severity of Depressive Symptoms

The 21-item Beck Depression Inventory (BDI) [24] was used, wherein a score of 0–10 indicates no or minimal depressive symptoms, 11–27 indicates moderate depressive symptoms, and 28 is cut for severe depressive symptoms.

### 2.3. Healthy Behavior Frequency

The Inventory of Healthy Behaviors [25] consists of four domains and each domain consists of six items as follows:

Domain 1. Recommended nutritional habits (fruit and vegetables, whole grain, sugar, salt, and cholesterol content of food, avoiding preservative food),

Domain 2. Preventive behaviors (health literacy, regular checkups, physical and mental hygiene),

Domain 3. Psychological attitudes (positive outlook, avoiding anger, anxiety, stress and depression, friendship, stable family life),

Domain 4. Health practices (avoiding overwork and excessive physical effort, sufficient rest and sleeping, controlling body weight, reducing tobacco consumption).

Respondents endorse the frequency of behavior in the last year on a 5-point scale, where 1 means “almost never”, 2—“rarely”, 3—“sometimes”, 4—“often”, and 5—“almost always.” The scores are converted into low, average, and high frequency of healthy behavior. The four domains can be assessed separately.

### 2.4. Statistical Methods

The statistical analyses were conducted using SPSS software. The mean (M) and standard deviation (SD) were estimated for continuous variables, as well as absolute numbers (n) and percentages (%) of the occurrence of items for categorical variables.

All estimates were calculated separately for rural women and separately for urban women.

The following statistical tests were used:

- Pearson’s chi-square test to compare the categorical variables between rural and urban women;
- Student’s t-test was used to compare continuous variables between rural and urban women, and to compare the severity of depressive symptoms and healthy behavior frequency, and to distinguish between the two categories of marital status and between the two categories of education level in urban women;
- F test of analysis of variance to compare the severity of depressive symptoms and healthy behavior frequency between the three categories of the level of education in rural women;
- Pearson’s correlation coefficient r to correlate the severity of depression and healthy behavior frequency with continuous characteristics.

We used both univariate and multivariate regression models of the severity of depressive symptoms against healthy behavior frequency. In univariate regression models, we correlated the severity of depressive symptoms with one domain of healthy behavior (covariate), while in the multivariate regression model we correlated the severity of depressive symptoms with four domains of healthy behaviors (covariates).

The significance level was assumed to be 0.05.

## 3. Results

### 3.1. General Characteristics of Rural and Urban Women

Postmenopausal women living in rural areas were significantly younger at last menstruation, more obese, less educated, more often widowed, and less often never married than urban women. There was no significant difference in age or motherhood (Table 1).

The same study group characteristics were presented in our previous study on the severity of depression in relation to serum TSH and vitamin D concentrations as well as metabolic syndrome [23].

### 3.2. Severity of Deprresive Symtoms—Comparison between Rural and Urban Women

Rural women were more likely to show moderate or severe depressive symptoms than urban women (*p* = 0.049, Table 2).

A negative correlation between the severity of depressive symptoms and age was found in postmenopausal urban women (*r* = −0.174, *p* = 0.029) but not in rural women (*r* = −0.034, *p* = 0.600). The older the urban postmenopausal women were, the less severe the depressive symptoms. The severity of depressive symptoms did not correlate with age at last menstruation, BMI, or the level of education, or marital status in postmenopausal women (Table 3).

### 3.3. Comparison of Health Behavior Frequency in Rural and Urban Women

Healthy behavior frequency was significantly lower in rural women, especially in Domain 1 (nutrition) (Table 4).

**Figure 1:**
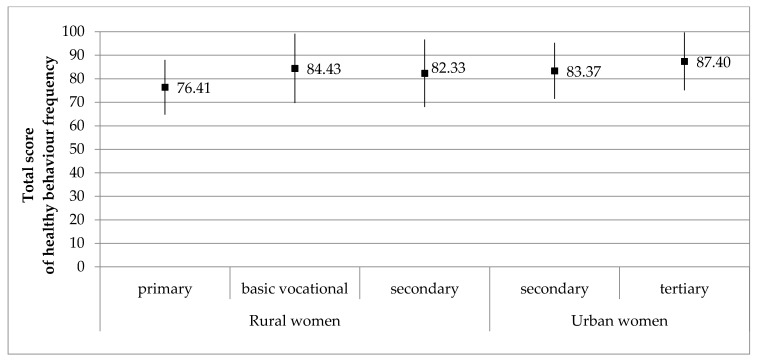
Total score of healthy behavior frequency with level of education in the study groups. Results are presented as M ± SD, M—mean, SD—standard deviation.

Healthy behavior frequency did not correlate with age, age at last menstruation, BMI, or marital status in postmenopausal women (Table 5). However, a correlation was found between healthy behavior frequency and the level of education in both rural and urban women (*p* = 0.034 and 0.045, respectively). Rural residents with a primary level of education had a significantly lower frequency of healthy behaviors than those with basic, vocational or secondary levels of education. Urban residents with a secondary level of education had a significantly lower frequency of healthy behaviors than those with higher education (Figure 1).

### 3.4. Correlation between Severity of Depressive Symptoms and Healthy Behavior Frequency in Rural and Urban Women

The severity of depressive symptoms correlated negatively with

- healthy behavior frequency, especially psychological attitudes in both rural and urban women;
- frequency of recommended nutritional habits and health practices only in urban women, but not in rural ones (Table 6).

## 4. Discussion

The present study demonstrated that in the case of rural women, the frequency of healthy behaviors, especially correct eating habits, was significantly lower and correlated with the level of education. The lower the education, the lower the frequency of healthy behaviors. In this group, as expected, the severity of depressive symptoms correlated negatively with the frequency of healthy behaviors and positive psychological attitudes. It means that with an increase in involvement in healthy behaviors and a positive attitude toward life, the severity of depressive symptoms reduces. Such a negative correlation between positive psychological attitudes and the severity of depressive symptoms is obvious, especially is cross-sectional studies. On the other hand, in the group of urban women, the higher the frequency of healthy behaviors—mainly aimed at adherence to a proper diet, showing an optimistic attitude to life and undertaking healthy practices—the lower the severity of depressive symptoms was.

Since menopause is an inevitable part of the aging process and marks the end of a woman’s reproductive years, it is associated with many unfavorable changes in the state of physical and mental health, which makes the research on disorders, diseases and their correlates of postmenopausal women essential [26].

A lower education level of postmenopausal women living in rural areas was observed in previous studies performed in various populations [22,27,28]. Studies based on Polish women demonstrated that the perimenopausal period more often predisposes rural women to changes in body structure, especially weight gain and changes in the distribution of adipose tissue [4,22,28]. The differences between rural and urban women were also reported in variables such as socioeconomic status and lifestyle. It is much more likely for rural women to be unemployed and without sufficient income, which may undoubtedly have an impact on their physical and mental health [22].

In the BDI, the surveyed postmenopausal women from rural areas more often had moderate (49.37%) or severe depressive symptoms (4.60%) when compared to urban women (42.68% and 1.27%, respectively). The present study showed that, apart from the place of residence, only age correlates with the severity of depressive symptoms, but only in urban postmenopausal women. The older the women were, the less severe were their depressive symptoms. Moreover, the severity of depressive symptoms did not correlate with any of the other variables studied (age of last menstruation, BMI, level of education, marital status). Similarly, in the Nigerian research based on 103 women aged 45–60 [29], no significant correlation was found between depressive symptoms and age, marital status, number of years after menopause and the level of education. Deveci et al. [30] observed that the mean BDI score of 519 postmenopausal women aged over 49 years old was 17.01 ± 8.75, and 42.2% had moderate and severe depression. Similarly, in a Spanish study [31], 45% out of 169 women suffered from depression. On the other hand, in studies conducted on groups of 744 Turkish women [32], 566 women from Taiwan [33], as well as women from southeast Poland (aged 48–58) analyzed by Barnaś et al. [34], a slightly lower proportion of postmenopausal women with depression compared to our study (24.7%, 38.7%, and 39%, respectively) was demonstrated.

The reduction of depressive symptoms with age was observed in 15 year longitudinal studies, however, women were not divided according to the place of residence [33,35,36]. Numerous data gathered so far have indicated that depression in postmenopausal women correlates with many socio-demographic variables, e.g., marital status, level of education, socioeconomic status.

Higher levels of depressive symptoms were observed in single, widowed, divorced, and unmarried women [2,21,37].

Women with lower levels of education were reported to have higher rates of depressive symptoms [2,14,30,32]. Low socioeconomic status was also a predictor of depressive symptoms [2,30]. Moreover, problems in sexual life, inactivity, and fertility/number of births per woman are important in the development of depression [30]. Previously, the age of first menstruation and the number of miscarriages were found as significant predictors of depression severity in the studies by Jung et al. [38], which was conducted in a sizable group of 60,114 Korean women aged 52–61, and the Chinese research by Zhang et al. [37] based on the contrary on a low number of women (N=51, aged 40–60).

Earlier studies have confirmed the relationship between the level of depressive symptoms and suboptimal healthy behavior. The severity of depression was associated with a lack of physical activity and a sedentary lifestyle in 13,715 women from 45 to 50 years of age [39]. Moreover, poor diet resulted in obesity correlated with the severity of depressive symptoms [33,40]. Smoking, excessive alcohol consumption, and poor sleep quality were other predictors of depressive symptoms in postmenopausal women [2,40,41].

The conducted analyses emphasize the importance of taking into account psychosocial factors, the place of residence, and the lifestyle of postmenopausal women to improve the quality of their life and health and reduce the risk of depressive symptoms. The research results also indicate the need for pro-health measures and preventive programs for postmenopausal women, which may inter alia compensate for the inequalities in the health of rural women. It is also important for women to be aware that their mental health in the fifth and subsequent decades of their lives depends on how much they care about their health. It seems justified to raise the level of disease prevention for women from rural areas, with access to prevention programs that usually are not available for farmers in Poland. A leading role in this process should be played by primary care practitioners, who should be involved in health promotion and health education.

The present study has some limitations. The proportion of urban/rural women in the present study differs from what is observed in the general population. Due to this fact, all analyses in the study were performed separately, so it did not affect the results. Despite the fact that the Beck Depression Inventory is often used in many studies, it includes questions about somatic symptoms that could be correlated with being overweight as well as with positive psychological attitudes and prevention behaviors. However, the last two domains are measured by the Inventory of Healthy Behaviors. Moreover, other factors can influence mood disorders, including psychological factors related to stress at work, at home, and in social relationships. The effects of these factors on depression severity in postmenopausal women are worth investigating.

## 5. Conclusions

The severity of depressive symptoms in this sample of Polish postmenopausal women correlates negatively with the frequency of healthy behaviors. The correlation is strongest in rural women.

## Figures and Tables

**Table 1:** Study group characteristics.

Variable, Parameter	Rural Women (*n* = 239)	Urban Women (*n* = 157)	Comparison between Rural and Urban Women	
			Test ^1^	*p*
Age (years), M ± SD	56.82 ± 4.40	56.38 ± 3.34	1.072	0.285
Age at last menstruation (years), M ± SD	48.95 ± 4.22	50.27 ± 4.08	−3.016	**0.003**
BMI (kg/m^2^), M ± SD	28.83 ± 5.01	26.36 ± 4.36	5.045	**<0.001**
normal weight	56 (23.43)	65 (41.40)	21.271	**<0.001**
overweight	96 (40.17)	64 (40.76)		
obesity	87 (36.40)	28 (17.83)		
Level of education, *n* (%)				
primary	27 (11.29)	1 (0.64)	168.621	**<0.001**
basic vocational	104 (43.51)	5 (3.18)		
secondary	97 (40.59)	65 (41.40)		
tertiary	11 (4.60)	86 (54.78)		
Marital status, *n* (%)				
married	204 (85.36)	128 (81.53)	24.504	**<0.001**
never married	3 (1.26)	7 (4.46)		
divorced	4 (1.67)	16 (10.19)		
widowed	28 (11.72)	6 (3.82)		
Having children, *n* (%)	228 (95.40)	144 (91.72)	2.251	0.134

^1^*t*-test for continuous variables or chi-square test for categorical variables, M—mean, SD—standard deviation. *p* for significant differences is in bold.

**Table 2:** Severity of depressive symptoms in study groups.

Depressive Symptoms	Rural Women (*n* = 239)	Urban Women (*n* = 157)	Comparison between Rural and Urban Women	
			*t* or Chi-Square Test ^1^	*p*
Total score, M ± SD	11.82 ± 8.43	10.29 ± 7.07	1.879	0.061
lack or minimal, *n* (%)	110 (46.03)	88 (56.05)	6.013	**0.049**
moderate, *n* (%)	118 (49.37)	67 (42.68)		
severe, *n* (%)	11 (4.60)	2 (1.27)		

^1^*t*-test for continuous variables or chi-square test for categorical variables, M—mean, SD—standard deviation. *p* for significant differences is in bold.

**Table 3:** Correlations between severity of depressive symptoms and study group characteristics.

Variable	Severity of Depressive Symptoms in Rural Women (*n* = 239)			Severity of Depressive Symptoms in Urban Women (*n* = 157)		
	Test		*p*	Test		*p*
Age (years)	r	−0.034	0.600	r	−0.174	**0.029**
Age at last menstruation (years)	r	−0.046	0.491	r	−0.103	0.207
BMI (kg/m^2^)	r	−0.002	0.976	r	0.106	0.186
Level of education	F (primary, basic vocational or secondary)	0.994	0.372	*t* (secondary vs. tertiary)	−0.357	0.721
Marital status	*t* (married vs. widowed)	−0.076	0.939	NA		

r—Pearson’s correlation coefficient; t-test to compare continuous variables between two groups; F test for analysis of variance to compare continuous variables between more than two groups; NA—not applicable because only the married group is large and the other groups are very small. *p* for significant differences or correlation is in bold.

**Table 4:** Healthy behavior frequency in study groups.

Healthy Behavior Frequency	Rural Women (*n* = 239)	Urban Women (*n* = 157)	Comparison between Rural and Urban Women	
			*t* or Chi-Square Test	*p*
Total score, M ± SD	82.46 ± 14.42	85.62 ± 12.13	−2.268	**0.024**
low, *n* (%)	88 (36.82)	38 (24.20)	7.062	**0.029**
average, *n* (%)	88 (36.82)	67 (42.68)		
high, *n* (%)	63 (26.36)	52 (33.12)		
Recommended nutritional habits, M ± SD	3.31 ± 0.73	3.51 ± 0.73	−2.697	**0.007**
Preventive behaviors, M ± SD	3.63 ± 0.74	3.76 ± 0.69	−1.772	0.077
Psychological attitudes, M ± SD	3.55 ± 0.72	3.65 ± 0.67	−1.481	0.139
Health practices, M ± SD	3.25 ± 0.74	3.34 ± 0.58	−1.244	0.214

*t*-test for continuous variables or chi-square test for categorical variables, M—mean, SD—standard deviation. *p* for significant differences is in bold.

**Table 5:** Correlations between total score of healthy behavior frequency and study group characteristics.

Variable	Total Score of Healthy Behavior Frequency in Rural Women (*n* = 239)			Total Score of Healthy Behavior Frequency in Urban Women (*n* = 157)		
	test		*p*	test		*p*
Age (years)	r	−0.169	0.009	r	0.166	**0.038**
Age at last menstruation (years)	r	0.039	0.568	r	0.154	0.058
BMI (kg/m^2^)	r	0.020	0.765	r	−0.073	0.366
Level of education	F (primary, basic, vocational or secondary)	3.436	**0.034**	*t* (secondary vs. tertiary)	−2.018	**0.045**
Marital status	*t* (married vs. widowed)	0.731	0.465	NA		

r—Pearson’s correlation coefficient; *t*-test to compare continuous variables between two groups; F test for analysis of variance to compare continuous variables between more than two groups; NA—not applicable because only the married group is large and the other groups are very small. *p* for significant differences or correlation is in bold.

**Table 6:** Regression models of severity of depressive symptoms on healthy behavior frequency in study groups.

Covariate	Severity of Depressive Symptoms in Rural Women (*n* = 239)				Severity of Depressive Symptoms in Urban Women (*n* = 157)			
	Univariate Models		Multivariate Model		Univariate Models		Multivariate Model	
	b	*p*	b	*p*	b	*p*	b	*p*
Total score of healthy behavior frequency	−1.89	**0.038**	NA	-	−5.10	**<0.001**	NA	-
Recommended nutritional habits	−0.96	0.205	−0.02	0.988	−2.91	**<0.001**	−0.98	0.221
Preventive behaviors	−0.95	0.202	1.02	0.341	−1.03	0.208	2.36	**0.007**
Psychological attitudes	−2.54	**0.001**	−3.58	**0.001**	−4.92	**<0.001**	−5.34	**<0.001**
Health practices	−0.70	0.346	0.66	0.473	−2.83	**0.004**	−0.91	0.357

b—mean change in depressive symptoms per unit of covariate; NA—not applicable because the total score of healthy behavior is calculated as a mean of four domain scores of healthy behaviors, so the total score is a linear function of four domain scores. *p* for significant slope terms is in bold.

## Data Availability

The data presented in this study are available on request from authors of the manuscript. The data are not publicly available due to privacy restrictions.

## References

[1] Harlow S.D., Gass M., Hall J.E., Lobo R., Maki P., Rebar R.W., Sherman S., Sluss P.M., de Villiers T.J. (2012). STRAW + 10 Collaborative Group. Executive summary of the Stages of Reproductive Aging Workshop + 10: Addressing the unfinished agenda of staging reproductive aging. Fertil Steril..

[2] Schneider H.P.G., Birkhäuser M. (2017). Quality of life in climacteric women. Climacteric.

[3] Tang R., Luo M., Li J., Peng Y., Wang Y., Liu B., Liu G., Wang Y., Lin S., Chen R. (2019). Symptoms of anxiety and depression among Chinese women transitioning through menopause: Findings from a prospective community-based cohort study. Fertil Steril..

[4] Zimny M., Starczewska M., Szkup M., Karakiewicz-Krawczyk K., Grochans E., Sipak-Szmigiel O. (2020). Analysis of the Impact of Type 2 Diabetes on the Psychosocial Functioning and Quality of Life of Perimenopausal Women. Int. J. Environ. Res. Public Health.

[5] Azizi M., Fooladi E., Masoumi M., Orimi T.G., Elyasi F., Davis S.R. (2018). Depressive symptoms and their risk factors in midlife women in the Middle East: A systematic review. Climacteric.

[6] Sassarini D.J. (2016). Depression in midlife women. Maturitas.

[7] Mauas V., Kopala-Sibley D.C., Zuroff D.C. (2014). Depressive symptoms in the transition to menopause: The roles of irritability, personality vulnerability, and self-regulation. Arch. Womens Ment. Health.

[8] Timur S., Sahin N.H. (2010). The prevalence of depression symptoms and influencing factors among perimenopausal and postmenopausal women. Menopause.

[9] Colvin A., Richardson G.A., Cyranowski J.M., Youk A., Bromberger J.T. (2017). The role of family history of depression and menopausal transition in the development of major depression in midlife women: Study of women’s health across the nation Mental Health study (SWAN MHS). Depress Anxiety.

[10] Lipińska-Szałek A., Sobczuk A., Pertyński T., Stetkiewicz T., Szymczak W. (2003). Biological and psychosocial factors influence on psychical issues of perimenopause. Przegląd Menopauzalny.

[11] Zalewska-Juzka A., Częstochowska E. (2003). Depresja w okresie okołomenopauzalnym. Pol. Merk. Lek..

[12] Bielawska-Batorowicz E. (2005). Menopausal symptoms in women and men aged 45–55. Przegląd Menopauzalny.

[13] Makara-Studzińska M., Wdowiak A., Bakalczuk G., Bakalczuk S. (2009). The effect of hormone therapy on the level of depression and quality of life in women in perimenopausal age, living in the countryside. Przegląd Menopauzalny.

[14] Bromberger J.T., Epperson C.N. (2018). Depression During and After the Perimenopause: Impact of Hormones, Genetics, and Environmental Determinants of Disease. Obstet Gynecol. Clin. N. Am..

[15] Worsley R., Bell R., Kulkarni J., Davis S.R. (2014). The association between vasomotor symptoms and depression during perimenopause: A systematic review. Maturitas.

[16] Ensan A., Babazadeh R., Aghamohammadian H., Afzal Aghaei M. (2018). Effect of Training Based on Choice Theory on Health-Promoting Lifestyle Behaviors among Menopausal Women. J. Midwifery Reprod. Health.

[17] Godycki-Cwirko M., Panasiuk L., Brotons C., Bulc M., Zakowska I. (2017). Perception of preventive care and readiness for lifestyle change in rural and urban patients in Poland: A questionnaire study. Ann. Agric. Environ. Med..

[18] Barros M.B.A., Lima M.G., Azevedo R.C.S., Medina L.B.P., Lopes C.S., Menezes P.R., Malta D.C. (2017). Depression and health behaviors in Brazilian adults—PNS 2013. Rev. Saude Publica.

[19] Peltzer K., Pengpid S. (2018). High prevalence of depressive symptoms in a national sample of adults in Indonesia: Childhood adversity, sociodemographic factors, and health risk behaviour. Asian J. Psychiatry.

[20] Pilewska-Kozak A.B., Dobrowolska B., Stadnicka G., Drop B., Jędrych M. (2019). Place of residence and age as variables differentiating health behaviours and perception of health by women past menopause. Ann. Agric. Environ. Med..

[21] (2006). Rural, Regional and Remote Health: Mortality Trends -2003.

[22] Kaczmarek M., Pacholska-Bogalska J., Kwaśniewski W., Kotarski J., Halerz-Nowakowska B., Goździcka-Józefiak A. (2017). The association between socioeconomic status and health-related quality of life among Polish postmenopausal women from urban and rural communities. Homo.

[23] Bojar I., Raczkiewicz D., Sarecka-Hujar B. (2020). Depression, Metabolic Syndrome, Serum TSH, and Vitamin D Concentrations in Rural and Urban Postmenopausal Women. Medicina.

[24] Beck A.T., Steer R.A., Ball R., Ranieri W.F. (1996). Comparison of Beck Depression Inventories -IA and -II in psychiatric outpatients. J. Pers. Assess.

[25] Juczyński Z. (2001). Measurement Tools in Health Promotion and Psychology.

[26] Soares C.N. (2017). Depression and Menopause: Current Knowledge and Clinical Recommendations for a Critical Window. Psychiatr. Clin. N. Am..

[27] Malacara J.M., Canto de Cetina T., Bassol S., González N., Cacique L., Vera-Ramírez M.L., Nava L.E. (2002). Symptoms at pre- and postmenopause in rural and urban women from three States of Mexico. Maturitas.

[28] Żołnierczuk-Kieliszek D., Kulik T.B., Jarosz M.J., Stefanowicz A., Pacian A., Pacian J., Janiszewska M. (2012). Quality of life in peri- and post-menopausal Polish women living in Lublin Province--differences between urban and rural dwellers. Ann. Agric. Environ. Med..

[29] Onya O.N., Otorkpa C. (2018). Biological and Environmental Correlates of Post-Menopausal Depression Among Gopd Attendees in Fmc, Lokoja, Nigeria. West Afr. J. Med..

[30] Deveci S.E., Açik Y., Dag D.G., Tokdemir M., Gündoğdu C. (2010). The frequency of depression and menopause-related symptoms in postmenopausal women living in a province in Eastern Turkey, and the factors that affect depressive status. Med. Sci. Monit..

[31] Pérez-López F.R., Pérez-Roncero G., Fernández-Iñarrea J., Fernández-Alonso A.M., Chedraui P., Llaneza P. (2014). MARIA (MenopAuse RIsk Assessment) Research Group. Resilience, depressed mood, and menopausal symptoms in postmenopausal women. Menopause.

[32] Unsal A., Tozun M., Ayranci U. (2011). Prevalence of depression among postmenopausal women and related characteristics. Climacteric.

[33] Wang H.L., Booth-LaForce C., Tang S.M., Wu W.R., Chen C.H. (2013). Depressive symptoms in Taiwanese women during the peri- and post-menopause years: Associations with demographic, health, and psychosocial characteristics. Maturitas.

[34] Barnaś E., Krupińska A., Kraśnianin E., Raś R. (2012). Psychosocial and occupational functioning of women in menopause. Menopause Rev./Przegląd Menopauzalny.

[35] Bromberger J.T., Schott L.L., Matthews K.A., Kravitz H.M., Harlow S.D., Montez J.K. (2017). Childhood socioeconomic circumstances and depressive symptom burden across 15 years of follow-up during midlife: Study of Women’s Health Across the Nation (SWAN). Arch. Womens Ment. Health.

[36] Mitchell E.S., Woods N.F. (2017). Depressed mood during the menopausal transition: Is it reproductive aging or is it life?. Womens Midlife Health.

[37] Zhang Y., Zhao X., Leonhart R., Nadig M., Wang J., Zhao Y., Wirsching M., Fritzsche K. (2019). A Cross-Cultural Comparison of Climacteric Symptoms, Health-Seeking Behavior, and Attitudes towards Menopause Among Mosuo Women and Han Chinese Women in Yunnan, China. Transcult Psychiatry.

[38] Jung S.J., Shin A., Kang D. (2015). Menarche age, menopause age and other reproductive factors in association with post-menopausal onset depression: Results from Health Examinees Study (HEXA). J. Affect. Disord..

[39] van Uffelen J.G., van Gellecum Y.R., Burton N.W., Peeters G., Heesch K.C., Brown W.J. (2013). Sitting-time, physical activity, and depressive symptoms in mid-aged women. Am. J. Prev. Med..

[40] Hickey M., Schoenaker D.A., Joffe H., Mishra G.D. (2016). Depressive symptoms across the menopause transition: Findings from a large population-based cohort study. Menopause.

[41] Amore M., Di Donato P., Papalini A., Berti A., Palareti A., Ferrari G., Chirico C., De Aloysio D. (2004). Psychological status at the menopausal transition: An Italian epidemiological study. Maturitas.

